# Macrophages and Metabolic Reprograming in the Tumor Microenvironment

**DOI:** 10.3389/fonc.2022.795159

**Published:** 2022-02-15

**Authors:** Jin Liu, Mingwei Gao, Zhou Yang, Yidan Zhao, Kun Guo, Binwen Sun, Zhenming Gao, Liming Wang

**Affiliations:** ^1^ Engineering Research Center for New Materials and Precision Treatment Technology of Malignant Tumors Therapy, Dalian Medical University, Dalian, China; ^2^ Engineering Technology Research Center for Translational Medicine, Dalian Medical University, Dalian, China; ^3^ Division of Hepatobiliary and Pancreatic Surgery, Department of General Surgery, The Second Affiliated Hospital of Dalian Medical University, Dalian, China

**Keywords:** immunology, macrophages, metabolic stress, tumor-promotion, intervention

## Abstract

Due to the emergence of traditional drug resistance in tumor treatment, the anti-cancer therapies are facing multiple challenges. Immunotherapy, as a new and universal treatment, has been gradually concerned. The macrophages, as an important part of the immune system, play an important role in it. Many studies have shown that immune state is essential in cancer progression and prognosis, rebuilding the architecture and functional orientation of the tumor region. Most tumors are complex ecosystems that change temporally and spatially under the pressure of proliferation, apoptosis, and extension of every cell in the microenvironment. Here, we review how macrophages states can be dynamically altered in different metabolic states and we also focus on the formation of immune exhaustion. Finally, we look forward to the explorations of clinical treatment for immune metabolism process.

## Introduction

Tumor-associated macrophages (TAMs) are important immune cells in the body, which constitute a plastic and heterogeneous cell population of the tumor microenvironment (TME) that can account for up to 50% of some solid neoplasms (1), play a vital role in anti-infection, immune surveillance and anti-tumor. Increasing evidence has shown that there is connection with energy regulation, immune surveillance and vital organ functions. Cancer changes the relationship with the immune state in various ways, such as the production of certain cytokines (2), antigen presentation (3), the immune response (4), checkpoint expression (5), and immune exhaustion (6). The function of immune cells depends on the normal process of nutrient metabolism. However, due to the hyperactive proliferation of tumor cells, the demand for nutrients is greatly increased. This also results in an imbalance in nutrient metabolism and supply between tumor cells and other stromal cells, which leads to immune cell dysfunction. The analysis of such a complex network can help to understand how organisms balance the energy expenditure of the immune response and the energy expenditure necessary for life; In turn, this may help identify new pharmaceutical targets of intervention in immune therapy focused on tumors.

## Two States of Macrophages Activation

Macrophages are extremely plastic polarized cells responding to stimulation from the tumor microenvironment and obtaining a specific functional phenotype. These cells are usually classified into proinflammatory (M1 macrophages) (7, 8) and anti-inflammatory (M2 macrophages) (9, 10). M1 macrophages are characterized by their ability to induce a proinflammatory response and present their antigens to T lymphocytes for the initiation of adaptive responses. Recently, various underlying transcriptional metabolic programs have been made to better define the process. Proinflammatory stimuli mainly induce the activation of transcription-specific pathways through the activation of transcription factors such as the nuclear factor kappa-light-chain enhancer of B-cells (NF-κB) (11, 12), signal transducer and activator of transcription 1 (STAT1) (13), STAT3 (14, 15), activator protein 1 (AP-1) (16), IFN-γ regulatory factor (IRF4) (17, 18), and hypoxia-induced factor 1 alpha (HIF1α), which triggers the expression of markers such as inducible nitric oxide synthase (iNOS), cyclooxygenase 2 (COX-2), CD80, CD86, and other major histocompatibility complex class II receptor (MHC-II) expression and the release of IL-1β, TNF-α, IFN-γ, IL-6, IL-12, and IL-23 rebuilding the immunological homeostatic control (19, 20).

M1 macrophages undergo metabolic reprogramming toward pentose-phosphate pathway flux relative to glycolysis enhancement, and fatty acid synthesis, which involves the Krebs cycle, ROS formation and citrate efflux (21). ROS and mitochondrial production stabilizes HIF-1α by activating NADPH, succinate and PGE2 synthesis (22). Itaconate is recognized to be involved in many immunosuppressive processes. Along with enhancing OXPHOS metabolism, fatty acid oxidation, tryptophan catabolism and glutaminolysis (23–25), M2 macrophages, which are characterized by the expression of CD163, CD206, adenosine receptor (A2R), and IL1RII (26), cooperate with resident cells for anti-inflammation, tissue regeneration, angiogenesis, tumor progression and metastasis (27) through the secretion of cytokines such as TGF-β, IL-1Ra, IL-4, IL-8, IL-10, IL-13, endothelial growth factor (EGF), and t (VEGFA) controlled by the activity of transcription factors STAT6, peroxisome proliferator-activated receptor gamma (PPARγ), and liver X receptor (LRX) (28).

## The Conversion of Macrophages

With the release of chemotactic and inflammatory factors in the tumor environment, blood-derived monocytes are recruited into tissue and differentiate into macrophages. At an early stage, these cells harbor an M1 phenotype with increased glycolysis, favoring tumorigenesis through proinflammatory functions. With the exuberance of tumor metabolism, such as the production of α-ketoglutarate (αKG) *via* glutaminolysis and the aggravation of hypoxia and engagement of fatty acid oxidation (FAO), macrophages likely switch to the M2 phenotype, leading to immunosuppressive functions favoring Th2 lymphocyte tissue infiltration and secretion of cytokines to promote tissue repair (29–31). M2 macrophages are always accompanied by UDP-GlcNAc-associated galactosylated modification prompting the glutamine pathway (29, 32).

## Macrophage and Cytokines

Cytokines act as mediators to regulate intercellular functions by affecting the activation of many immune cells and promoting the differentiation of tumor-associated subtypes. Hypoxia and glucose metabolism favor neoangiogenesis. Endothelial cell recruitment relies on TAM-derived products, including vascular endothelial growth factor A (VEGFA), VEGF receptor 2, adrenomedullin, angiopoietin 2, C-X-C motif chemokine ligand 8 (CXCL8), and CXCL12 (28, 33, 34), which, respectively, were observed both *in vitro* and *in vivo* in human and breast cancer models in mouse (35). In turn, M2-like macrophages promote tumor deterioration by secreting growth factors, proangiogenic molecules (EGF, VEGFA), immunosuppressive factors (IL-6, IL-10, TGF-β, iNOS), and proteases that remodel the extracellular microenvironment such as matrix metallopeptidases (27, 36, 37).

Fat metabolism plays an important role in macrophages immune function. The suppression of FAO favored M2 transmission to the M1 phenotype, which was observed in murine models of lung and colorectal cancer (38). MIF secreted by cancer cells supports pulmonary carcinogenesis through the upregulation of fatty acid synthase (FASN) in specific murine TAMs (39). In addition, FABP5 is highly expressed in TAMs infiltrating breast cancer, which is associated with lipid droplet accumulation and secretion of immunostimulatory cytokines, including IL-6 FABP4 is upregulated in macrophage infiltration in late-stage breast cancer, which supports tumor progression by favoring IL-6 expression through NFκB-miR-29b pathway signaling (40).

**Figure 1:**
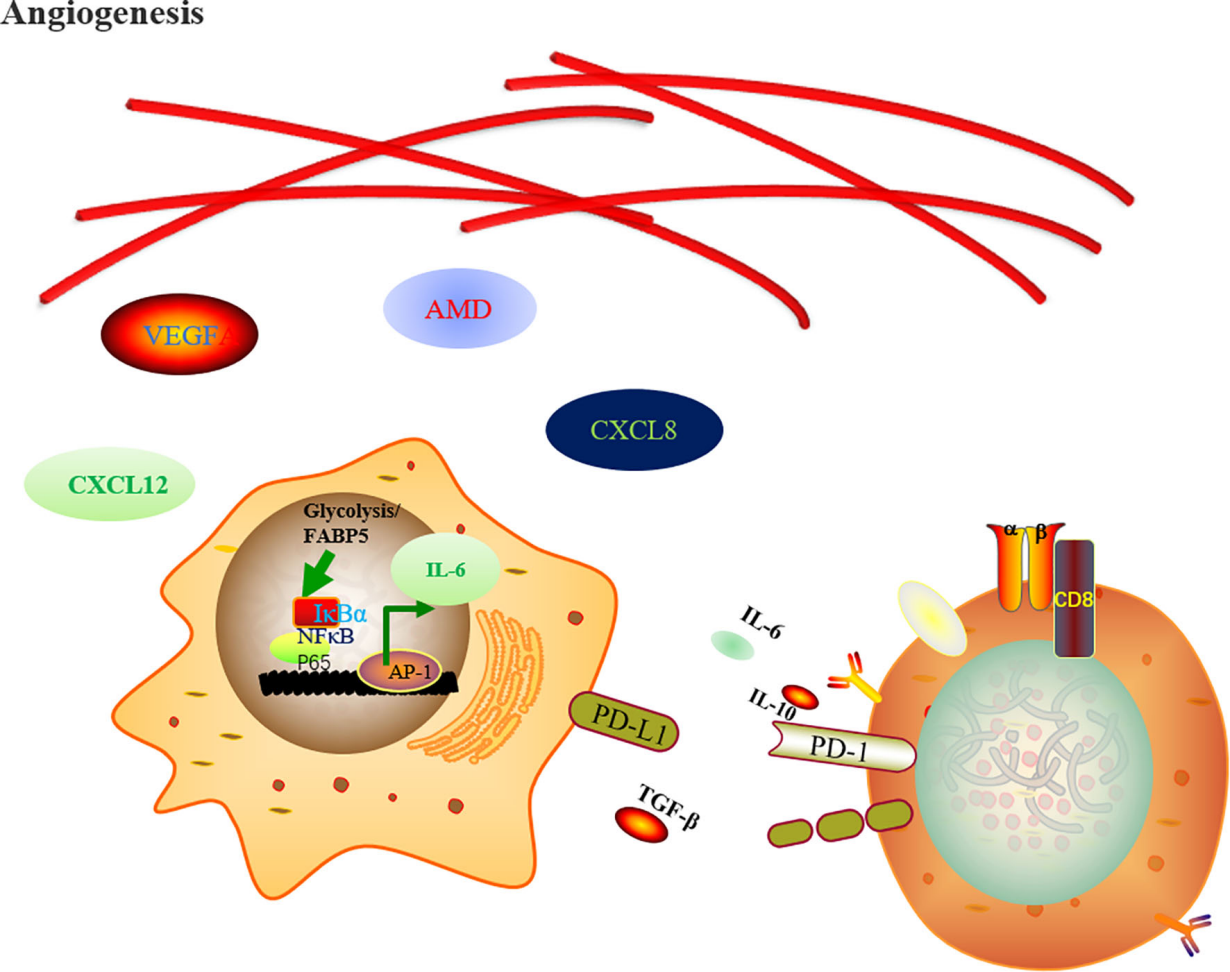
Regulation of metabolic rewiring upon macrophage cells activation. The TAM-derived hypoxia and glucose metabolism products favor neoangiogenesis through endothelial growth factor A (VEGFA), adrenomedullin (AMD), C-X-C motif chemokine ligand 8 (CXCL8), and CXCL12. In the meantime, the FABP5 and the metabolite of glycolysis favor the tumor progression by expression of IL-6 and PD-L1 through NF-κB pathway signaling inhibiting CD8+ T cells anti-tumor effects.

Besides that, chemotherapeutic stress, oxygen tension, a lack of nutrients and the accumulation of metabolites promote the release of CSF1, VEGF, and IL34 to reeducate TAMs, favor neoangiogenesis and vascular dysfunction and decrease antigen presentation (41, 42). TAMs secrete IL-6 to favor glycolysis by promoting phosphoglycerate kinase 1 (PGK) phosphorylation, which favors aerobic glycolysis and tumorigenesis (43) (**Figure 1**).

## Macrophages Serve as Antigen Presenting Cells

Generally, dendritic cells are thought to perform antigen cross-presentation to activate CD8+ T lymphocytes. Recently, some other immune cells such as macrophages founded to be capable of cross-presentation. Macrophages in the liver are very heterogeneous cell population which are also called as Kupffer cells (44, 45) play a major role in the clearance of the hepatic portal vein’s antigens and pathogens derived from the gut, making the liver ideally an immunologically privileged site. Macrophages are also been found to have the ability to stimulate medullary sinus macrophages inducing antitumor effects through stimulation of tumor-specific CD8+ T lymphocytes (46). These findings may explain why tumor-infiltrating macrophages in the tumor microenvironment are capable of cross-presentation (47, 48). The general control nonrepressible 2 (GCN2), a kinase activated by amino acid starvation, participates in macrophages killing intracellular pathogens and enhancing antigen presentation to CD4+ and CD8+ T cells (49–52) through mTOR pathway activation (53, 54). From recent studies, it has become evident that a clear picture of many pathogens, from viruses to multicellular parasites, have developed methods to alter the mTOR signal of macrophages to rebuild their metabolism and immune functions, thereby avoiding immune responses. These changes range from the control of cell metabolism to the regulation of protein expression and autophagy (55). The accumulation of metabolites provided transcriptional production of the inflammatory cytokines TNF and IL-6 through inflammatory epigenetic reprogramming modifications, including histone-acetyl transferases and methylation (56).

## Metabolism and Immune Response

Lactate and itaconic acid, produced by enhanced glycolysis, contribute to the establishment of immunosuppressive TME *via* various mechanisms, including extracellular acidification, resulting in functional energy of cytotoxic T and NK cells and reducing the recruitment of T cells (57). H^+^ and O_2_ are metabolites of glycolysis and key players in the tumor metabolic microenvironment (58–60). Both H^+^ and O_2_ play a dual role in tumor metabolism. The levels of metabolic products and reactants may not only influence reaction flux and thermodynamics but also regulate the expression and activity of important metabolic enzymes (61, 62).

Mitochondrial citrate is closely related to the production of various critical pro-inflammatory mediators in macrophages, including NO, ROS, and prostaglandin E2 (PGE2) and some others (62–64). Some evidence suggests that changes in adenosine deaminase (ADA) activity, particularly its ADA2 isoenzyme, are related to the development of malignant breast cancer (BC). The metastatic potential and recurrence of triple-negative breast cancer (TNBC) patients also demonstrated that the plasma ADA2 activity correlates positively with tumor M2 macrophage markers as well as the ADA1 activity correlates positively with endothelial dysfunction or inflammatory parameters (65). Adenosine signaling mediates several inhibitory functions of effector immune cells. Beyond that, PPARs are reported elicit responses essential to the physiological control of immune homeostasis, hematopoiesis, inflammation, development and metabolism, which constituting a promising target for the development of novel therapeutic interventions.

## The Exhaustion of the Immune System

The tumor microenvironment (TME) consists of not only malignant but also endothelial, stromal, and immune cells. Such complex interaction often involves extracellular metabolites, which constitute a source of energy supply as well as acting as communication signals between different cellular compartments thereby rebuilding it. Moreover, metabolic byproducts can also hijack the process.

Aerobic glycolysis induces PD-L1 expression on monocytes *via* the PFKFB3 NF-κB pathway (66). IGF-2 preprograms pro-inflammation stimulating macrophages that maintain the mitochondrial complex V activities elevating programmed death-ligand 1 (PD-L1) expression, which alleviated the inflammatory disease in the mice (67). mTOR may modulate osteoclastogenesis and reduce bone metastases in tumor-bearing mice by regulating the relationship between macrophages and T cells and the expression several of cytokines and costimulatory membrane receptors such as cytotoxic T-lymphocyte protein 4 (CTLA-4), programmed death 1 (PD-1) and so on (68). Nick P. Goplen et al. observed that myeloid deficiency of PPAR-γ resulted in selective impairment of the tissue-resident alveolar macrophage (AM) compartment, which acts as a negative regulator of tissue resident memory CD8 T cell (TRM) differentiation during influenza virus infection (69). CSFR1 blockade in macrophages enhanced the reactive response to anti–PD-1 treatment by increasing tumor surveillance by CD8 T+ cells (70). Nabiul et al. found that Na/H exchanger 1 (NHE1) is involved in the transformation of TAMs and PD-1 checkpoint activation of T cells in glioma. Blocking NHE1 function may restore glucose metabolism of immune cells necessary for promoting the efficiency of anti-PD-1 therapy (71). Keto et al. performed 50 markers of the cancer-immunity cycle in 101 patients with an RNA sequencing assay, which showed that high expression of checkpoints TIM-3 and VISTA and of the macrophage-associated marker CD68 were associated with significantly worse PFS after anti-PD-1/PD-L1-based therapies (72). TIM-3, in turn, acts on macrophages, and TIM-3 was demonstrated to negatively regulate the production of reactive oxygen species (ROS) and influence downstream proinflammatory cytokine secretion of IL-1β and IL-18 in macrophages *in vitro* (73).

## Clinical Practice of Macrophages Therapies

TAM is increasingly being recognized as a potential tumor therapeutic target in view of its various important roles in cancer progression.

Firstly, two strategies were proposed, one of it is based on the depletion or neutralization of M2-like TAMs. Another is shifting the balance from M2-type to M1-type TAMs. Then an anti-macrophage migration inhibitory factor (anti-MIF) antibody was applied to inhibit the chemotaxis of TAMs in solid tumors. 

In the early 20th century, the important relationship between metabolism and macrophages was gradually recognized. A number of promising therapeutic treatments are being developed to interrupt the immunometabolic crosstalk between malignant cells and TAMs (**Table 1**). 17β-Estradiol (E2) regulates the induction of chemokines and cytokines, such as interleukin 6 (IL-6), which modulates macrophage immune phenotypes. Furthermore, E2 enhances the adhesion of human umbilical vein endothelial cells (HUVECs) to various matrix proteins and increases cell migration, thus promoting angiogenesis mediated by estrogen receptor α activation (74, 75). Conjugated estrogens (CE)/bazedoxifene (BZA) therapy was applied to ductal breast carcinoma in *in situ* patients. To determine whether CE/BZA will modulate some aspects of immune function, a switch to an M2-type protumorigenic macrophage signature. TAMs with elevated glycolytic gene transcript levels were found to shape a prometastatic vascular net and augment extravasation of tumor cells breaking through the vascular endothelial basal membrane with higher levels of EMT (76). Besides that, anti-VEGFA or anti-ANGPT2 (Angiopoietin peptides 2), aiming at not only suppresses neoangiogenesis but also inhibit glycolysis in TME (77). *In vivo* mouse tumor models, oxidative metabolism was activated in hypoxic TAMs coupled with glucose intake decreasing, in the meantime, oxidative damage culminating in endothelial cell leading to angiogenesis and metastasis through the upregulation of DNA damage inducible transcript 4 (DDT4, also known as REDD1), an endogenous inhibitor of mechanistic target of rapamycin (MTOR) complex 1 (MTORC1) (78). Antiangiogenic drugs elicit hypoxia-triggered recruitment of myeloid cells, which may mediates resistance to current antiangiogenic therapies. Thus, targeting macrophage metabolism has potential complementary effects with antiangiogenic therapies in terms of tumor progression and tolerance to conventional therapies (79–81). Simvastatin was able to repolarize tumor-associated macrophages (TAMs), promoting M2-to-M1 phenotype conversion by targeting lipid metabolism to overcome EMT-associated drug resistance *via* the integrin β3/FAK pathway (82).

**Table 1:** The action under clinical development as of October 1st, 2021, for oncological indications; source, http://www.clinicaltrials.gov.

Mode of action	Condition	Progress	Status	Developer
β-glucan	Squamous Cell Carcinoma of the Oral Cavity	N/A	Active, not recntiting	Shih-Jung Cheng
Compound Vitamin Bl2	Nasopharyngeal Cancers	Phase II	N/A	WeiLUO
Fluvastatin sodium	Breast Cancer	Phase II	Completed	Laura J. Essennan
Migrat ion Inhibitory Factor (Anti-Mff) Antibody	Metastatic Adenocarcinoma of the Colon or Rectum	Phase I	Completed	Takeda
Pravastatin	Hepatocellular Carcinoma	Phase II	Terminated	Shehnaz Hussain
IDO1 Inhibitor	Oral Cavity Squamous Cell Carcinoma	Phase II	Recruiting	Adam Luginbuhl
β-glucan	Non Small Cell Lung Cancer	N/A	Recruiting	Goetz H Kloecker
Metfonnin Pioglitazone Hydrochloride	Oropharyngeal Neoplasm	Phase II	Terminated	Frank G Ondrey
Metfonnin Hydrochloride	Lung Carcinoma	Phase II	Not yet recruiting	Saikrishna S Yendamu ri
Estrogens/Bazedoxifene	Ductal Breast Carcinoma	Phase II	Recruiting	Swati Kulkarni

N/A, not applicable.

Considering the important effect of glycolysis and its metabolites on the immune function of anticancer therapy, many agents have been developed aiming at reducing lactate availability for glycosis in TME, deletion of LDHA (83) as well as the administration of 2-deoxyglycose (84), mTORC1-targeting agents (85) phosphatidylinositol-4,5-bisphosphate 3-kinase catalytic subunit gamma (PIK3CG/PI3Kγ) plus PIK3CD inhibitors (which jointly cause PKM2 downregulation) (86).

Monoclonal antibodies targeting PD-L1 or PD-1 are approved by the US Food and Drug Administration for the treatment of various solid tumors, known as immune checkpoint blockers (ICBs). Some studies have found that glucose metabolism is associated with PD-1 expression in series of cells (87, 88). Aerobic glycolysis induces PD-L1 expression on monocytes *via* the PFKFB3 NF-κB pathway (63). The contribution of tumor hypoxia to the resistance of all cancer therapies is well documented, including conventional therapy such as surgery, chemotherapy, radiotherapy, and recently therapy, immunotherapy. Antioxidant and glycolytic inhibitors should be combined with ICB therapies to improve the efficacy. In addition, CSF1 was found secreted accompany with T cells activated by PD-1-targeting ICBs (89). These observations provide strong reasonable ground for the combined PD-1 blocked and anti-CSF1R therapy (90).

**Figure 2:**
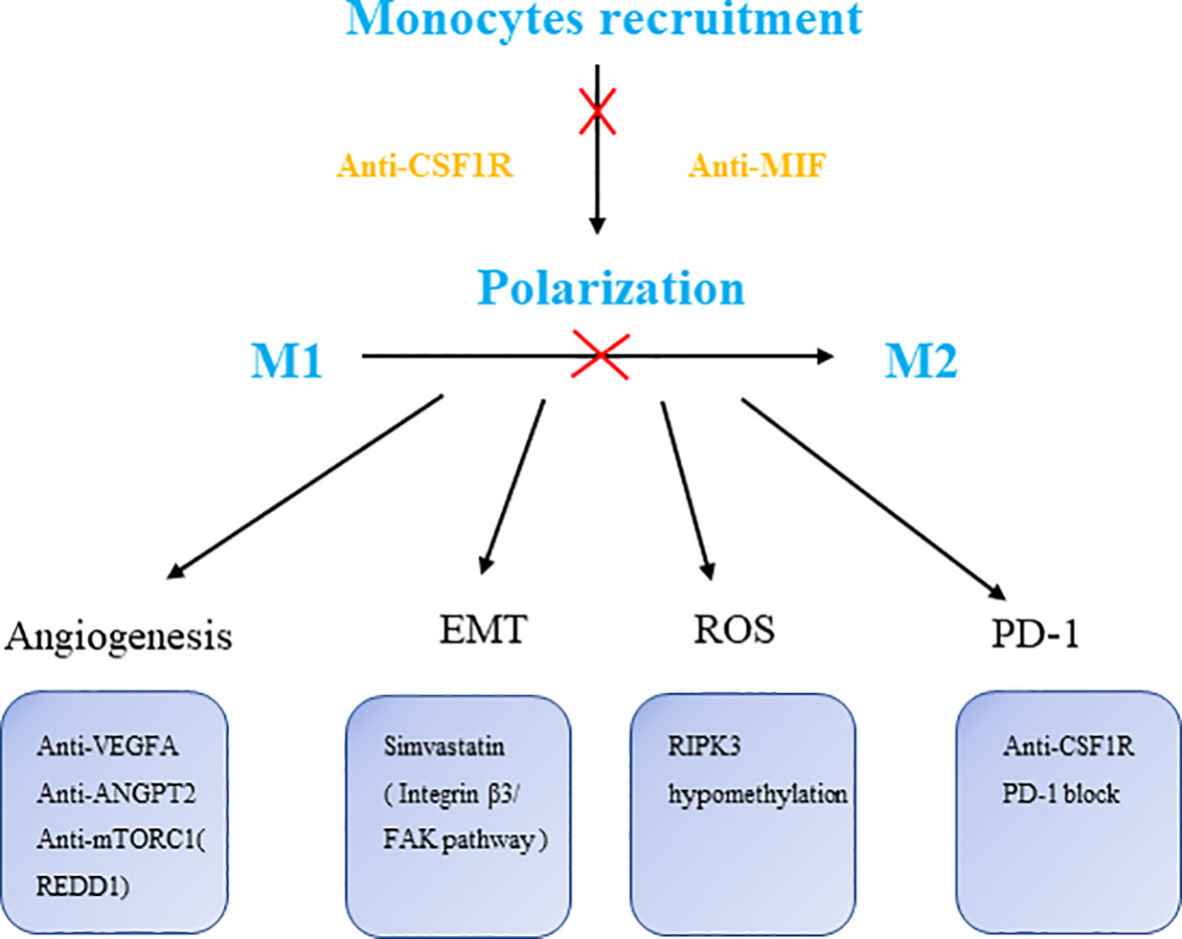
The targets of macrophage therapies. CSF1R, colony stimulating factor 1 receptor; MIF, macrophage migration inhibitory factor; VEGFA, vascular endothelial growth factor A; ANGPT2, angiopoietin-2; RIPK3, receptor-interacting protein 3.

Numerous biological processes performed by macrophages are controlled by the mTOR pathway, including metabolism, chemokine and cytokine production, antigen presentation, autophagy and survival (91).The mTOR signaling is associated with the development and progression of many cancers (92, 93), mTOR inhibitors have therapeutic value in a number of cancers in both preclinical and clinical settings (94). Of note, combining antagonists of PTEN and mTOR reduces cancer cell proliferation and improves clinical outcomes in 50% of glioblastoma patients (95). Interestingly, mTOR activation is antitumoral in hypoxic TAMs but protumoral in cancer cells. REDD1 seems to participate in enhancing glycolysis in KO TAMs, leading to glucose competition (GC), stabilizing tumor epithelial cell junctions and vessels and preventing metastasis. In those patients who do not respond, cancer cells remain sensitive to rapamycin *in vitro*, suggesting that a possible future study could focus on REDD1+ TAMs in response to drug-resistant treatments. Metabolic programming in tumor-induced macrophages shows an increase in glycolysis as well as higher ROS levels due to increased mitochondrial oxidation. Lei Wu et al. found that RIPK3 (Receptor-interacting protein 3) reprograms fatty acid metabolism in TAMs by the ROS-PPAR pathway. This signaling axis revealed that ROS represented by peroxide reactions also participate in the function of macrophages, thereby new therapeutic strategies centered on antioxidant peroxides were proposed (96) (**Figure 2**).

## Discussion

The investigation of TAMs has made great progress since the 1970s TAMs were confirmed to be the real host cells rather than the tumor cells.

However, there are still many key questions to be clarified about the relationship between TAMs and tumors. We must recognize that bioenergetic metabolism displays a high degree of heterogeneity across different TAM subpopulations as well as similar ones (97, 98). The suboptimal efficacy of current TAM-targeting approaches at least partly caused by these phenomenon (99). However, the relationship between TAM and tumor remains to be clarified: (1) With the development of studies in mononuclear phagocyte lineage, the mechanism of proliferation needs to be reexamined, especially source and maintenance levels of macrophages in human cancers. (2) The integrity of the tumor and the surrounding environment and heterogeneity and subject characteristics, all cells in the environment of *in situ* research are particularly important in time and space. Different space should be more widely studied to observe the change in the cells during different stages with the overall objective of examining the occurrence of tumor development. In recent years, single-cell transcriptomics and spatial transcriptomics have made it possible to study a large number of cellular components in spatial-temporal dimensions. However, due to the high cost of research, the research is limited to a small number of specimens. (3) To what extent macrophages represent the immune microenvironment of the tumor. How does the diversity of TAM within or between different kinds of tumor. It is not clear whether these reflect intrinsic organizational characteristics or host fitness response. (4) Although, it has long been known that high levels of TAM are associated with poor prognosis in many human tumors, however, TAM’s assessment is not used as a definitive and widely used benchmark in pathological grading evaluation, a series of high-quality clinical studies targeting macrophages in multiple tumor types are urgently needed.

## Author Contributions

JL, MG, ZY, YZ, and LW developed the main concept and designed the study. JL completed the literature collection, the article writing, and the drawing of all the pictures and tables. MG, ZY, and YZ assisted with the literature collection part of the manuscript and edited it. KG, BS, and ZG provided guidance and the proofreading of the article. LW supervised the whole process, provided suggestions for the article writing. All authors contributed to the article and approved the submitted version.

## Funding

This work was supported by the fund from the National Natural Science Foundation of China (81972749 and 81471755), Science and Technology Program of Liaoning Province (2018225056), Dalian innovation fund project (2018J12SN085), and Department of Education of Liaoning Province (XLYC1802011).

## Conflict of Interest

The authors declare that the research was conducted in the absence of any commercial or financial relationships that could be construed as a potential conflict of interest.

## Publisher’s Note

All claims expressed in this article are solely those of the authors and do not necessarily represent those of their affiliated organizations, or those of the publisher, the editors and the reviewers. Any product that may be evaluated in this article, or claim that may be made by its manufacturer, is not guaranteed or endorsed by the publisher.
